# A novel portable anechoic chamber using ultra-thin 2D microwave absorber for industrial 5.0

**DOI:** 10.1038/s41598-024-55595-4

**Published:** 2024-03-04

**Authors:** Amit Kumar Baghel, Youssef Bikrat, Joana Tavares, Henrique Chaves, Vinicius Uchoa Oliveira, Pedro Pinho, Nuno Borges Carvalho, Helena Alves

**Affiliations:** 1 Instituto de Telecomunicações, 3810-193 Aveiro, Portugal; 2 INESC MN, Instituto Superior Técnico, 1049-001 Lisbon, Portugal; 3 LES, PHYSIC DÉPARTEMENT, MOHAMMED 1st OUJDA, 60000 Oujda, Morocco; 4 Universidade de Aveiro and Instituto de Telecomunicações, 3810-193 Aveiro, Portugal

**Keywords:** Electrical and electronic engineering, Design, synthesis and processing, Characterization and analytical techniques, Electrical and electronic engineering, Design, synthesis and processing, Characterization and analytical techniques

## Abstract

In this paper, the authors, for the first time, have shown the use of 2D conformal microwave absorbing material (MAM) in the design and fabrication of a portable Anechoic chamber (AC). The MAM is fabricated on the transparent and conductive metal oxide layer named indium-tin-oxide (ITO) with Polyethylene terephthalate as the substrate and the ground plane for zero transmission having overall thickness of 0.012$\lambda$ where $\lambda$ is calculated at 0.7 GHz. The MAM is characterized for 0.7 to 18 GHz for both TE- and TM-polarisation and oblique incidence. High sheet resistance, dipole-like resonance structure patterned on the ITO, and the air-spacing between the layers is optimized to achieve broadband absorption. The MAM is used to line the six sides of the rectangular anechoic chamber having inner dimensions of: (*L* × *W* × *H*: 850 × 650 × 720 mm^3^). The return loss (*RL*), gain, and radiation pattern of three antenna working at 1.56, 2.43, and 4.93 GHz are analyzed inside the AC. The measurement results for all frequencies very well match with the simulation studies, thus validating and opening the door for the future use of ultra-thin and planar MAM in the AC.

## Introduction

Absorbers, as the name suggests, are used either to absorb the sound^1^ or electromagnetic waves^2^ to avoid unwanted reflections from the rigid body, thus giving lower electric-field ripple^3^ or noise. In this paper, the electromagnetic wave absorber for microwave frequency is discussed. The microwave absorbing material (MAM) is used for different applications, like in aircraft to reduce the radar cross-section and make them difficult to track by the enemy radar, designing anechoic chambers (AC) to avoid unwanted reflections from external objects and provide free-space antenna characterization and radar cross-section measurement^4^, electromagnetic interference/electromagnetic compatibility solutions for shielding purposes^3,5–9^ are proposed. Different materials like polyurethane, polyethylene, and expanded polystyrene are used commercially as the absorbing material for AC^3^. However, commercially available absorbers are bulky, fragile, and thick. This reduces the test zone for the antenna under test and limits the electrical size of the antenna to be characterized. Industrial revolution 5.0 demands advanced materials to make them economical and ecologically sustainable^10^. Polyurethane foam is made up of isocyanates, which are hazardous to health^11^.

The MAM can be classified broadly into two categories according to the band of operation, *i*.*e*.,  narrow-band and broadband. The narrow-band MAM structure was first introduced by Landy et al. in 2008^12^, where two different resonators are proposed for electric and magnetic resonance. After the proposed design, other narrow designs like Salisbury screen^13^, Jauman screen^14^, polarisation insensitive dual band absorber^15^, and Dallenbach screen^16^ are proposed. The split maze is proposed to get resonance at different frequencies in X (8-12 GHz) and Ku (12-18 GHz) bands in^17^. The use of super-formula to propose the frequency-selective surface to have narrow-band resonance at 3.5 GHz is shown in^18^. All the above-proposed designs are narrow-band and thus have lower practical utility. To increase the bandwidth, two approaches are taken. The first one is the use of ferrite materials, graphite-based carbon nanoparticles and fiber, and silica-coated iron micro flakes in absorption phenomena due to their exceptionally good magnetic property, chemical stability, and stability of the oxidation resistance even in high temperature^19–26^. The higher temperature MAM is designed using antimony-doped tin dioxide (ATO)/($SiO_2$) micro-spheres are used to achieve a maximum reflectivity of − 47.3 dB at 573 K^23^. However, the achieved bandwidth is 2.4 GHz. An overview of using carbon and its composites to design MAM is presented^24^. The requirement of having higher microwave absorption properties at a lower cost is difficult to achieve in the present scenario with the trade-off between the two. The use of transition metal carbides ($Ti_3C_3T_x$ MXene or $Mo_2TiC_2$MXene microfibers) in MAM requires complex and costly refining processes and cannot be used for large-scale applications like in the construction of the AC^25,26^. Also, the effective absorption bandwidth is lower, thus making it unsuitable for applications requiring broadband absorption. The use of the ferrite material approach requires the controlled mixing of various chemicals to fabricate the end material. This makes the process complex and requires suitable expertise. The other approach is to use high resistive sheets^27–29^ and multi-layer resonant structure^30,31^ to have higher losses at the surface and resonant frequencies of each metallic layer structure close to each other, respectively. The higher losses cause a lower quality factor and thus increase the absorption bandwidth. However, the commercially available resistive sheets with the required sheet resistance ($R_s$) can be used in the second approach. The use of lumped resistance for broadband absorption from 13.42 to 22.66 GHz is proposed^32^. However, the performance will degrade if one of the lumped elements stops working. Also, optimization of the design to get the required performance takes a large amount of computational time. The use of origami to increase the bandwidth to 24.6:1 is proposed^33^. The proposed design is 3-dimensional (3D), and the likelihood of performance getting degraded while using the AC due to braking of the edges is very high. Recently, research into the design of absorbers for the visible regime has also gotten traction. The use of a three-layer absorber based on Manganese (Mn)-silica (SiO_2_)-Manganese (Mn) is proposed, where a square disk is surrounded by a square ring made of Mn for 447 to 717 nm^34^. The experimental validation of the above is still to be seen. The second approach for the broadband MAM is used in this paper, considering the ease of fabrication and material availability.

Having analyzed the literature on MAM, none of the presented literature tried to use the MAM to design AC and characterise the antenna. The authors intended broadband MAM for AC and antenna characterization in this paper. The use of ultra-thin MAM for the AC will help to increase the internal quiet zone (QZ) volume for the exact outer dimensions of the AC compared to conventional 3D-MAM. As the proposed MAM is planar and has no sharp tips, the problem of breaking and degrading its performance easily due to human lapses will be minimized. This will help in the long-term durability of the proposed AC. The MAM is fabricated using a resistive sheet made of indium-tin-oxide (ITO) with polyethylene terephthalate (PET) as the substrate. The $R_s$ with the Styrofoam spacer is optimized to give broadband absorption. The polarization insensitive dipole-like structure is etched on the ITO to give a resonance around 6.5 GHz. The optimized MAM is placed on the side walls of the AC, and the antenna radiation pattern for three microstrip antennas working at 1.56, 2.43, and 4.93 GHz is measured. The structure of this paper is as follows: After the introduction, the second Section gives the design theory and optimization of the MAM. The fabrication process for the MAM is provided in “Fabrication and characterisation of MAM” section. The gain uncertainty calculation of the AC with MAM using two antenna methods is given in “Gain uncertainty calculation of AC with the MAM” section. The fifth section gives the antenna characterisation where the return loss (*RL*), the radiation pattern, and gain are measured inside the proposed AC and the conventional AC. Finally, the conclusion is made in the final section.

## Design theory and optimization

**Figure 1:**
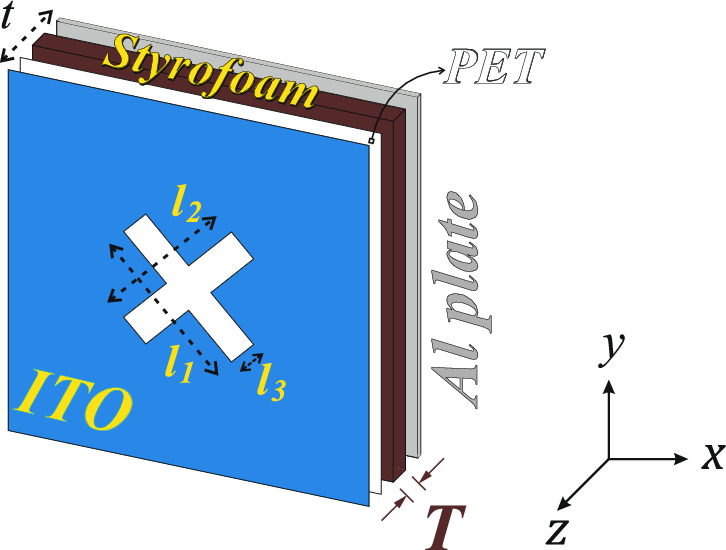
MAM unit cell showing different layers (optimized parameters: *p* = 50 mm, $l_{1}$ = $l_{2}$ = 22 mm, $l_{3}$ = 5 mm, *T* = 4.2 mm, and *t* = 5.4 mm).

**Figure 2:**
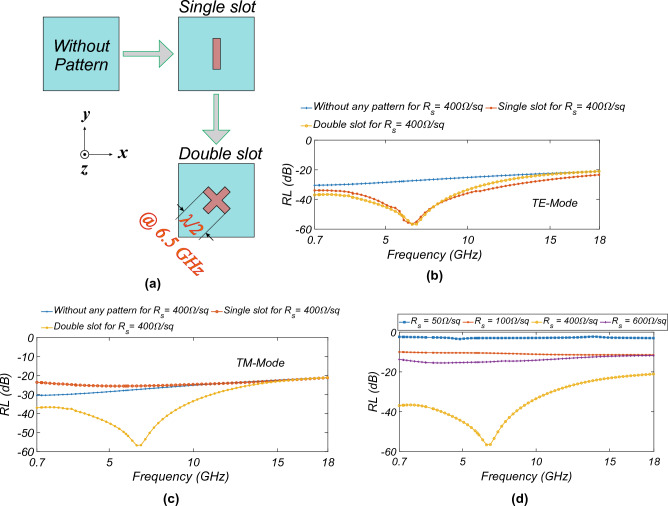
(**a**) Schematic of different iteration for the design, *RL* for (**b**) TE-, (**c**) TM-mode excitation for the different iteration, and (**d**) variation of *RL* with the $R_{s}$.

Figure 1 shows the proposed MAM having different layers. The top conductive layer is made of ITO+PET with a thickness of 0.2 mm ($e_{rPET}$ = 3.25, $tan\delta$ = 0.02). For broad-band performance, Styrofoam (STY) as the spacer is used ($e_{rSTY}$ = 1.11). To get the perfect absorption, an aluminum (*Al*) metal plate with 1 mm thickness (considering the practical thickness available) is used at the bottom of the proposed MAM. The unit cell’s periodicity (*p*) is chosen to be 50 mm to have the dipole-like resonance of the slot at 6.5 GHz. The ITO conductive layer with the optimized $R_s$ (in $\Omega /sq$) is etched on the PET. The 45° cross is etched to have a fourfold symmetry and a performance independent of the polarization. The different iterations of the design to arrive at the final design are shown in Fig. 2a. The simulation studies use computer simulation technology (CST) in a finite difference frequency domain setup with unit cell boundary conditions. The *RL* for $R_{s}$ = 400 $\Omega /sq$ in all the cases is shown in Fig. 2b and c for TE- and TM-mode, respectively. The figure shows that without the pattern, there is no resonance, and the *RL* is due to the resistive nature of the ITO sheet. With the single slot, the resonance in TE mode is only seen due to the *y*-polarised electric field exciting the slot. The TM mode is not excited due to the lower height of the slot in the *x* direction. In the case of a double-slot design, the *RL* for the TE- and TM-modes is the same because of the fourfold symmetry of the design with the same height of slot in *x*- and *y*-direction. The cross-structure is chosen to have a larger ITO surface area, thus having more effective sheet resistance, and to have dipole-like resonance at 6.5 GHz (see Fig. 2a). Figure 2d shows the variation of the $R_s$ with the frequency. The *RL* is > − 20 dB for smaller $R_s$. With the optimized $R_s$ and slot dimensions, the advantage of the resonance and resistive nature of ITO is taken to get *RL* $\le$ − 20 dB.

**Figure 3:**
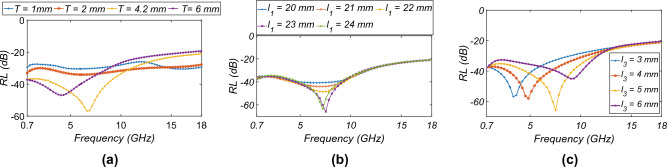
*RL* variation with different parameters: (**a**) *T*, (**b**) $l_{1} = l_{2}$, and (**c**) $l_{3}$.

**Figure 4:**
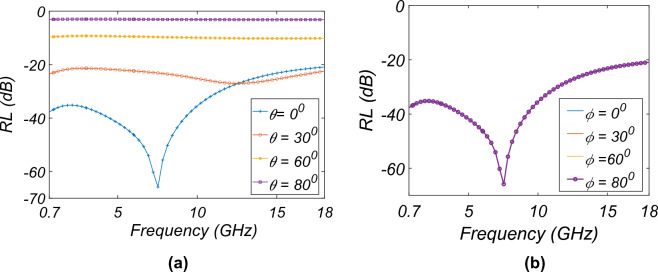
*RL* variation with $\theta$ and $\phi$ for TE/TM: (**a**) $\theta$, (**b**) $\phi$.

**Figure 5:**
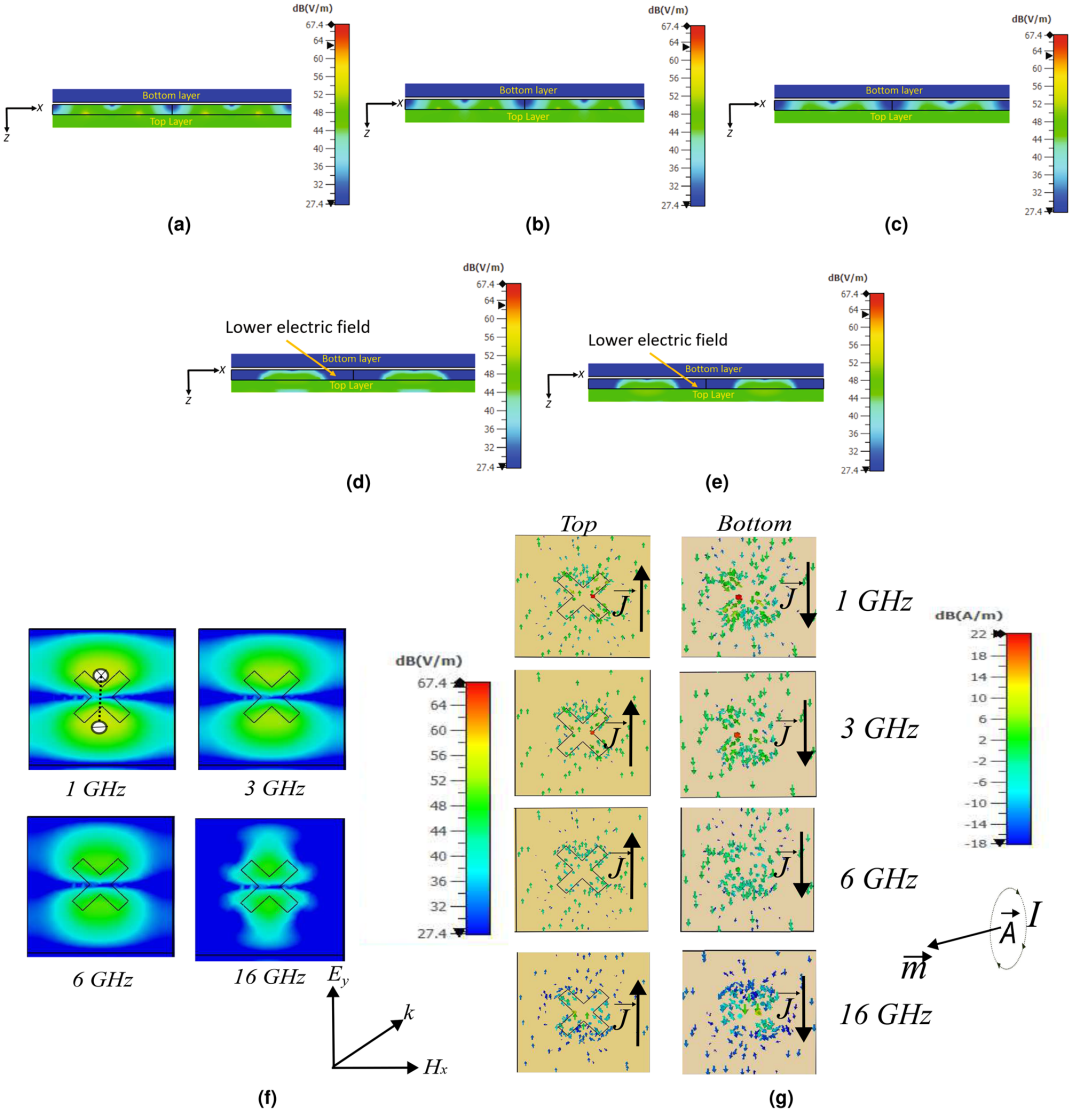
Electric field inside the substrate of the MAM at: (**a**) 1 GHz, (**b**) 3 GHz, (**c**) 6 GHz, (**d**) 12 GHz, and (**e**) 16 GHz . (**f**) electric field on top surface, and (**g**) $\vec {J}$ at different frequency.

**Figure 6:**
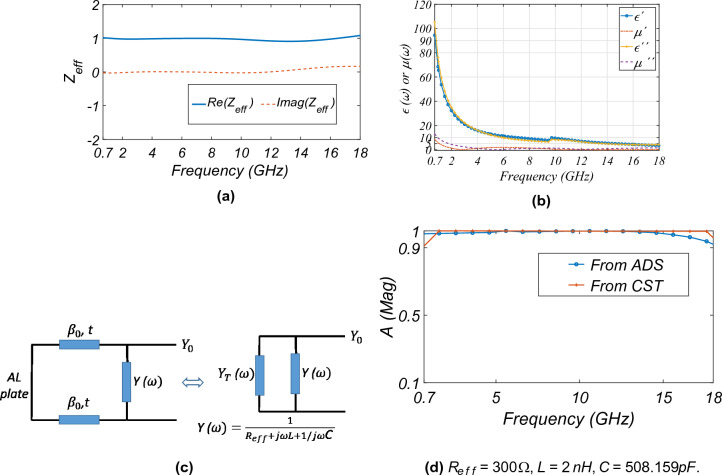
(**a**) $Z_{eff}$ variation with frequency, (**b**) the effective $\epsilon (\omega )$ and $\mu (\omega )$, (**c**) equivalent circuit, and (**d**) comparison of absorption with the equivalent circuit from ADS and finite difference frequency domain analysis in CST.

The variation of the *RL* with the thickness of the spacer (*T*), the height ($l_{1}$), and the width of the slot ($l_{3}$), keeping the $R_{s}$ = 400 $\Omega /sq$, is shown in Fig. 3. From Fig. 3a, the *RL* is strongly lower than -40 dB below the frequency of 6 GHz for the *T* = 4.2 mm. This is because $T\,<\,\lambda$/4 for frequencies lower than 6 GHz; thus, the resonance behavior is observed for frequencies lower than 6 GHz. Figure 3b shows the variation of the $l_{1}$ with frequency. For $l_{1}$ = 23 mm, the resonance is seen around 6 GHz because of the half-wavelength dipole behavior of the slots ($\lambda /2$ = 25 mm at 6.5 GHz). When increasing the $l_{3}$, the resonance peak shifts to a higher frequency because of lower capacitive behavior between the two ends of the slot, as seen in Fig. 3c. The behavior of the MAM for the different incidence ($\theta$) and polarisation angle ($\phi$) for TE and TM-mode is shown in Fig. 4. The TM plot for the different $\theta$ and $\phi$ is not shown separately as the behavior of MAM is the same as shown by the TE-mode plots and to preserve the brevity of the manuscript. Figure 4a shows that the performance till $\theta$ = $60^0$ is acceptable with $RL \le$ − 10 dB. As the MAM is fourfold symmetric, the *RL* for different $\phi$ variations is the same as observed from 4b. The loss in the MAM is due to three processes: (1) power dissipation in the dielectric; (2) conduction loss; and (3) impedance matching with the free space. To understand the absorption phenomenon due to the power dissipation in the dielectric, the electric field inside the substrate is plotted in Fig. 5a–e. The average power dissipation density ($P_d$ = $\dfrac{|E |^2 \omega \,tan\delta \varepsilon ^{'}}{2}$, where $\varvec{E}$ is the maximum value of the electric field ($\vec {E}$), $\omega$ is the frequency of operation, and $\epsilon ^{'}$ is the real part of the effective value of electrical permittivity, and $\tan \delta$ is the loss tangent of the dielectric^35^). The figure shows that the intensity of the electric field up to 6 GHz is higher and more homogeneously spread throughout the substrate, thus utilizing the whole volume of the substrate for the loss. However, after 6 GHz, the electric field is concentrated in the middle of the substrate. The absorption lowers, and maximum absorption happens due to resistive ITO on the top surface. This is also true as the thickness of the substrate becomes more than $\lambda /4$ around 8 GHz, and the absorption happens at the surface^27^.

The $\vec {E}$ at the top surface and the $\vec {J}$ at the top and bottom surfaces of the MAM are shown in Fig. 5f and g, respectively. The electric dipole moment ($\vec {p}$ = $q\vec {d}$, where *q* is the charge at the opposite sides of the branches of MAM and $\vec {d}$ is the distance between them) in the direction of the field is developed and decreases at the higher frequency,*i*.*e*., 16 GHz. Similarly, due to the anti-parallel $\vec {J}$ at the top and bottom surfaces of the MAM, an effective magnetic dipole moment ($\vec {m}$ = $\vec {A}I$ where $\vec {A}$ and *I* are the effective area of the loop and current, respectively) is created. The simultaneous creation of $\vec {p}$ and $\vec {m}$ at the lower frequencies helps in increasing the conduction loss. Also, it is observed that the effect of the same decreases at higher frequencies due to lower $\vec {J}$. The normalized effective input impedance *w.r.t* free space ($Z_{eff}$) is shown in Fig. 6a. The $Z_{eff}$ is given by^19^:

1 $$\begin{aligned} Z_{eff}(\omega ) = \sqrt{\dfrac{\mu (\omega )}{\varepsilon (\omega )}} =\sqrt{\dfrac{(1+S_{11}(\omega ))^2-S_{21}(\omega )^2}{(1-S_{11}(\omega ))^2-S_{21}(\omega )^2}} \end{aligned}$$

where, $\mu (\omega )$ = $\mu ^{'}(\omega ) - i\mu ^{''}(\omega )$ is the effective magnetic permeability, $\varepsilon (\omega )$ =$\varepsilon ^{'}(\omega ) - i\varepsilon ^{''}(\omega )$ is the effective electrical permittivity with real ($\mu ^{'}(\omega )$,$\varepsilon ^{'}(\omega )$) and imaginary term ($\mu ^{''}(\omega )$,$\varepsilon ^{''}(\omega )$), $S_{11}(\omega )$ is the reflection coefficient, and $S_{21}(\omega )$ is the transmission coefficient ($S_{21}$ = 0 as the MAM is backed by the conducting plate). To have perfect absorption and zero reflection $\sqrt{\dfrac{\mu (\omega )}{\varepsilon (\omega )}}$ is adjusted to give $Z_{eff}(\omega )$ $\approx$ 1, which is evident from Fig. 6a having an imaginary part close to zero, thus having perfect absorption. Figure 6b shows the values of $\varepsilon (\omega )$ and $\mu (\omega )$^36^. It is observed that the larger values of $\varepsilon ^{'}(\omega )$ and $\mu ^{'}(\omega )$ until 8 GHz cause higher losses due to the formation of strong electric and magnetic dipoles, as evident from the earlier discussions (see Fig. 5g). Figure 6c shows the equivalent circuit where $\beta _{0}$ is the free-space phase constant, *t* is the overall combined thickness of the substrate, spacer, and aluminum, $Y_{T}(\omega )$ is the effective admittance of the short circuit line at the input port, and $Y(\omega )$ is the effective admittance due to the series effective resistance ($R_{eff}$), inductance (*L*) due to the ITO conductive layer, and capacitance (*C*) due to the spacing between the length of the dipole elements. $R_{eff}$ is due to $R_{s}$ and the ohmic losses. The coupling capacitance between the two unit cells is minimal due to the larger gap between the slots on the ITO and is ignored for simplicity. This will not affect the equivalent circuit derived from the CST simulations. The absorptivity (A($\omega$, $\theta$)) is given by^37^:

2 $$\begin{aligned} A(\omega , \theta ) = 1- R(\omega ,\theta )- T(\omega ,\theta ) \end{aligned}$$

where $R(\omega ,\theta )$ and $T(\omega ,\theta )$ are the co- and cross-polarised reflected and transmitted power, respectively. Figure 6d shows the $A(\omega )$ magnitude from the CST and the equivalent circuit drawn in the Keysight Advanced Design System (ADS). The figure shows that the absorption magnitude well matches the unit cell simulation results. The value of the lumped elements is given in the caption of the figure.

## Fabrication and characterisation of MAM

**Figure 7:**
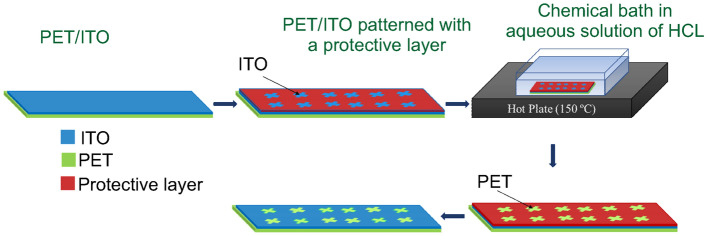
PET/ITO chemical etching process fabrication.

ITO on PET substrate was patterned using a shadow mask and a protective layer. The pattern was developed in a 5-min chemical bath in an aqueous solution of HCl (37$\%$, Across Organics) at 150 °C. The developer was removed with distilled water, followed by acetone, in an ultrasound bath for 1–5 min. The sample was immersed in a water solution with a nonionic detergent in ultrasounds for 5 min. The patterned piece was then washed with acetone HPLC, acquired from Enzymatic, in ultrasounds for 5 min, followed by an additional 5 min immersed in 2-propanol from Enzymatic. The fabrication process is exposed in Fig. 7.

**Figure 8:**
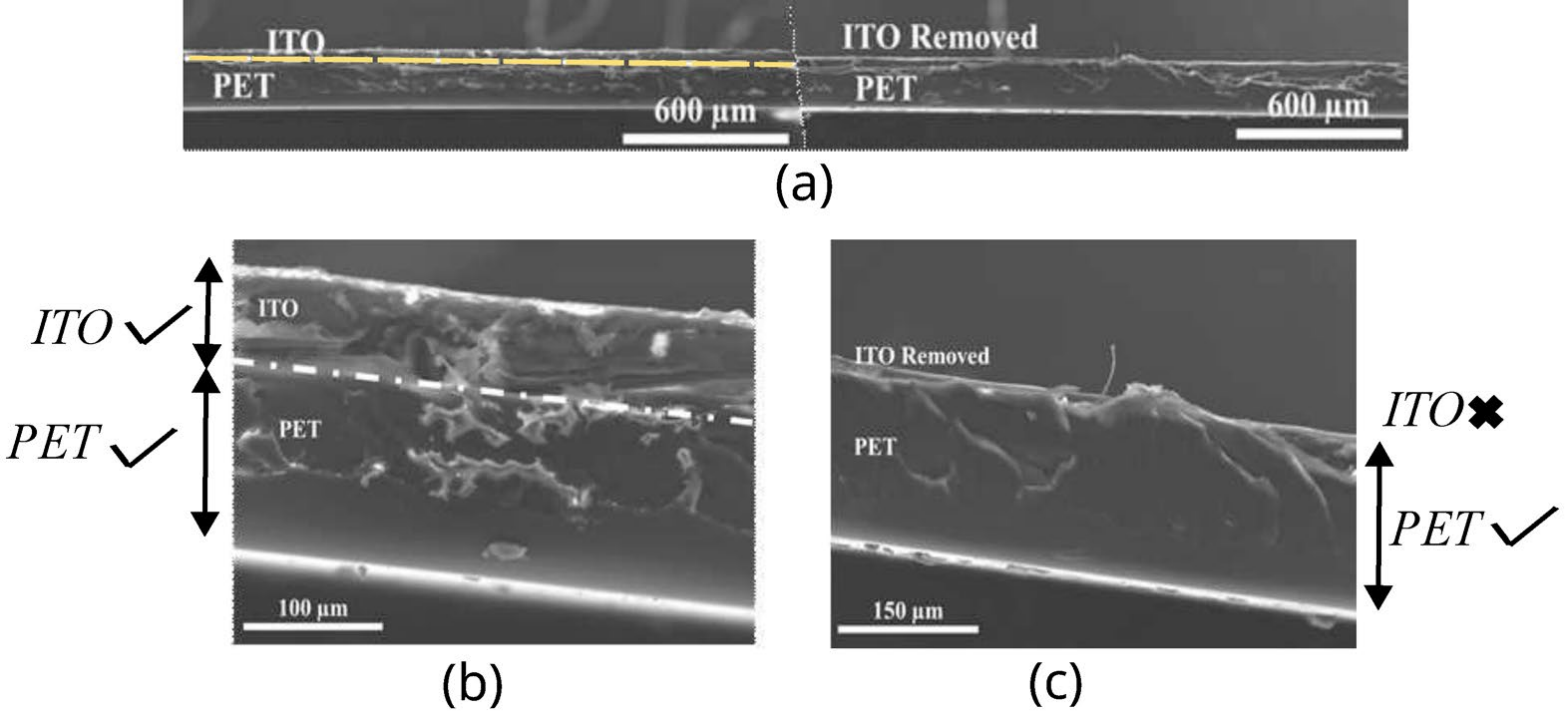
Transversal SEM images: (**a**) zoom-out, zoom-in: (**b**) PET/ITO before chemical etching, and (**c**) PET/ITO after chemical etching.

To analyze the effect of etching on PET/ITO thickness, morphology, and the effectiveness of the etching process, a scanning electron microscopy (SEM) evaluation was performed on a Hitachi S4100. A transversal analysis of a PET/ITO sample before and after the chemical etch was implemented (Fig. 8a). A 0.19 mm and $0.6\,\upmu \hbox {m}$ thickness was observed for PET and ITO, respectively, with an overall substrate thickness of 0.2 mm (Fig. 8b). After etching, the protected layers kept the thickness and morphology, while they were effectively eliminated in unprotected areas (Fig. 8c). This proves that the fabrication process successfully removed the intended ITO.

**Figure 9:**
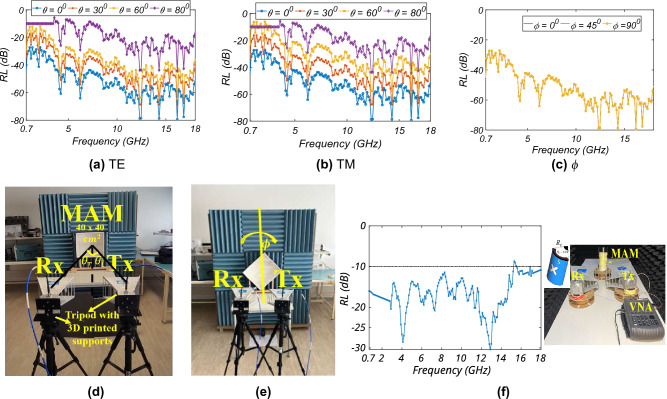
Results and measurement setup: (**a**) TE, (**b**) TM, (**c**) polarisation, measurement setup for different: (**d**) $\theta$, (**e**) $\phi$, and (**f**) conformality measurement.

The performance of the MAM is observed by measuring the *RL* for both the normal and oblique incidence in TE and TM polarisation. Also, for measuring the *RL* for different polarisation, the MAM is rotated at a discrete angle. The MAM array measuring 40 × 40 cm^2^ is taken. A metal plate of the same dimensions is taken to normalize the measurements and to remove any reflection effects from the surrounding environment. The MAM is kept in the far field of the transmitting (Tx) and receiving (Rx) antennas. Figure 9a and b show the measured *RL* for TE and TM polarisation for different $\theta$, respectively. It is observed that the performance degrades for a higher oblique incidence angle after $\theta$ = 60°. This is because as the incident field grazes through the surface, it sees less of the absorber surface. This phenomenon will also be observed while measuring the radiation pattern at $\theta$ = $90^0$, where there is some deviation in the simulated and measured results. Figure 9c shows the *RL* for $\phi$ variation. It is observed that the *RL* overlaps with different $\phi$ similar to the simulation result; thus, the proposed MAM is polarisation insensitive. Figure 9d and e show the measurement setup to measure the *RL* for $\theta$ and $\phi$ variation, respectively. To check the performance of the MAM for conformality, the MAM is bent with the central angle $\theta _{c}$ given by $\frac{180.p}{\pi .R}$, where *R* is the radius of curvature. The MAM is wrapped around the cylindrical, curved object, with *R* calculated for $\theta _{c}$ of $120^0$. Figure 9f shows the measured *RL* for the same. Also, the measurement setup is shown at the right of the figure. The figure shows that the *RL* for $\theta _{c}$ = $120^0$ is lower than − 10 dB throughout the band; thus, the proposed MAM also gives stable performance while bending. This is useful in placing the MAM at the rectangular corners to achieve lower *RL*.

**Figure 10:**
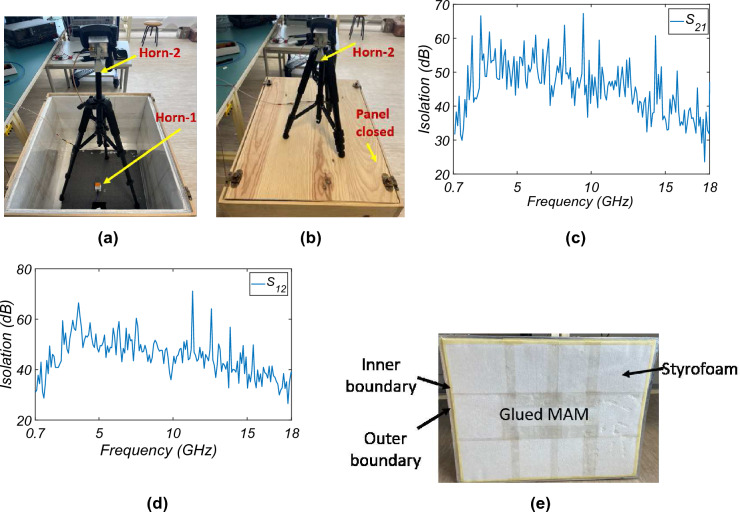
Different setup for isolation measurement (**a**) panel open, (**b**) panel closed. Isolation measurement data: (**c**) from inside to outside (**d**) outside to inside, and (**e**) top panel .

**Figure 11:**
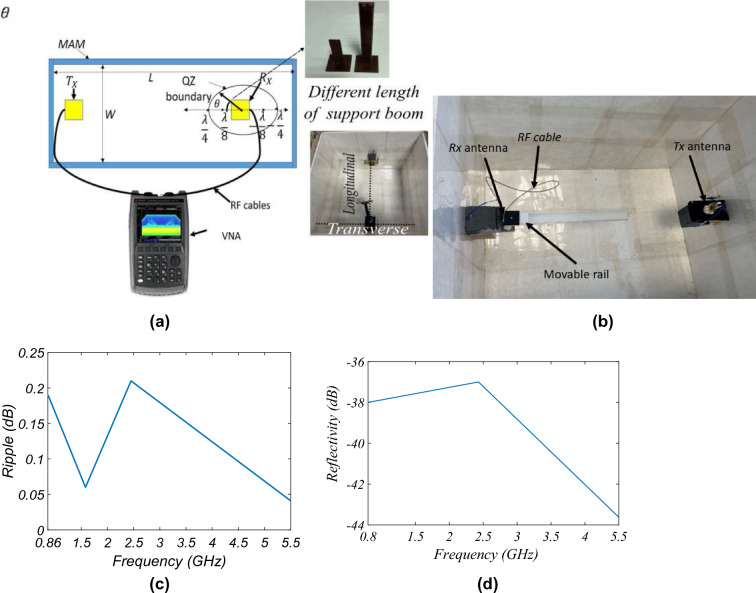
Ripple measurement: (**a**) schematic and (**b**) actual measurement setup, (**c**) measured ripple in bore-sight, (**d**) reflectivity.

## Gain uncertainty calculation of AC with the MAM

After the testing of the MAM for different polarisation, angles, and conformality, the AC with the MAM is characterized by performing different standard tests. The AC has internal dimensions of 850 × 650 × 720 mm^3^. The MAM is stuck to the interior walls of the AC. The 4 mm thick Styrofoam with a 1 mm aluminum plate is used as the spacer and shielding purpose, respectively. The outer casing is made of wood and has wheels at the bottom to ease moving from one place to another. The uncertainty in the measured gain due to different instruments and sources is summarized below.

### Instrumentation uncertainty

 Many factors affect the measurement of reflection ($S_{11}$) and transmission coefficient ($S_{21}$) from the programmable network analyzer (PNA). These are PNA noise floor and non-linearity, calibration standard repeatability, cable bending, and tightening uncertainty. These uncertainties can be found in the manufacturer’s data sheet and uncertainty calculator. The measurements are done after 30 min to avoid thermal drift of the PNA electronic components. The PNA receiver linearly will affect the measurement process, and its uncertainty can be measured^38^.

### Isolation test

 For the isolation test, the procedure described in IEEE Standard 299 and EN50147-1 is followed. For this, the transmission coefficient for inside to outside ($S_{21}$) and outside to inside ($S_{12}$) is calculated for two case scenarios: (1) when the chamber panel is open, and (2) when the chamber panel is closed. Figure 10a and b show the measurement setup for measuring the isolation for the open and closed panels, respectively. Here, port-1 is defined for the antenna inside the chamber and port-2 for the antenna outside the chamber. The measurement is done from 0.7 to 18 GHz. The ratio of $S_{21}$ or $S_{12}$ measured with the chamber panel open to the chamber panel closed gives the isolation level. Figure 10c and d show the isolation measured from inside to outside and outside to inside, respectively. For good isolation, the top panel, as shown in Fig. 10e, is provided with a stepped layer, i.e., two boundaries: an inner edge, which goes inside the AC and gives the required shielding, and the outer boundary, which is attached to the AC outer casing with the help of circular rivets.

### Ripple measurement

 Figure 11a shows the schematic and the setup to measure the ripple and reflectivity in the QZ. As the proposed AC is designed for the test purpose of demonstrating the feasibility of using MAM as an alternative to the conventional absorber, the ripple in the QZ having a maximum diameter of $\lambda /2$ is done, where $\lambda /2$ is the minimum frequency of measurement, i.e., 0.86 GHz. The movable platform is displaced from the central place in the steps of $\lambda /8$ on both sides (in the direction of transmitting (Tx) antenna and away from it) with the receiving (Rx) antenna kept on it as shown in Fig. 11b. Figure 11c shows the measured ripple from 0.86 GHz to 5.5 GHz. The measurements are done at this range of frequencies because of the device under test (DUT) and to respect the far-field conditions according to the physical dimensions of the antenna and the test setup. It is observed that the maximum average ripple is 0.12 dB, which is an acceptable limit for gain measurement. To characterize the QZ for various transverse directions, the reflectivity test is done according to the voltage standing wave ratio method^39^. The receiver antenna is rotated with respect to the longitudinal directional with different supporting boom lengths (to vary the transverse distance, see inset Fig.11a). The maximum reflectivity measured with respect to different frequencies is given in Fig. 11d. The pattern measurement uncertainty due to the QZ reflectivity is ± 0.1 dB, which is within the range of 0.12 dB as taken in the gain uncertainty calculation^39^.

### Distance and polarisation uncertainty

 The distance between Tx and Rx (*r*) is kept constant in all the antenna gain measurements and is measured using 8 m stainless steel tape with the measurement uncertainty given in^40^. For the polarisation uncertainty measurements, the cross-line laser from Bosch is used for vertical and horizontal alignment.

### Phase error

 To have a phase uncertainty of 0.1 dB, the antenna has to be placed at least at $r_{min}$ = $\frac{4D^2}{\lambda }$, where *D* is the maximum dimension of the DUT in the case of the same Tx and Rx antennas^41^. Table 1 shows the $r_{min}$ value for different DUT. The table confirms that the DUT is always kept at *r* > $r_{min}$, as *r* is 59 cm in this case.

**Table 1:** $r_{min}$ of different antenna for 0.1 dB phase uncertainty.

DUT test frequency (GHz)	*D* (mm)	$r_{min}$ (mm)
1.56	99	203
2.43	118	452
4.93	58	228

### Mismatch in calibration

 The mismatch uncertainty (*u*) in the calibration is given by^42^:

3 $$\begin{aligned} u = \frac{|\Gamma _{gen}|.|\Gamma _{receiver}|. |S_{21}|. |S_{21}|}{\sqrt{2}} \end{aligned}$$

where $\Gamma _{gen}$ and $\Gamma _{receiver}$ are the absolute values of the reflection coefficient values of the generator and receiver sides, respectively. $|S_{21}|$ and $|S_{12}|$ are the gains from the generator to the load and from the load to the generator, respectively. For the calibration at the ports of the antennas, $|S_{21}|$ = $|S_{21}|$, respectively. The $|\Gamma _{gen}|$ and $|\Gamma _{receiver}|$ values are found in the manufacturer data sheet^43^.

Considering the different sources of uncertainty in the antenna gain (*G*) measurement and their respective values, the combined and expanded uncertainty values with $95\%$ confidence level are tabulated in Table 2. An expanded standard uncertainty in the measured gain due to a different source of error is 0.578 dB. It will be shown later that the measured *G* is within the range of this uncertainty.

**Table 2:** *G* uncertainty budget using two antenna method.

Source	Reference	Value (dB or %)	Distribution	Divisor	Standard uncertainty (dB)
Receiver linearity (reference measurement)	Data sheet	0.01	Rectangular	1.732	0.005
Receiver linearity (DUT measurement)	Data sheet	0.12	Rectangular	1.732	0.005
Ripple (reference measurement)	Measurement	0.12	Normal (k=1)	1	0.12
Ripple (DUT measurement)	Measurement	0.12	Normal ((k=1))	1	0.12
Polarization error (reference measurement)	Data sheet	0.05%	Rectangular	1.732	0.001
Polarization error (DUT measurement)	Data sheet	0.05%	Rectangular	1.732	0.001
Phase error (reference measurement)	Data sheet	0.1	Rectangular	1.732	0.01
Phase error (DUT measurement)	Data sheet	0.1	Rectangular	1.732	0.001
Mismatch in calibration (reference measurement)	Data sheet	0.001	Normal (k=1)	1	0.001
Mismatch in calibration (DUT measurement)	Data sheet	0.001	Normal (k=1)	1	0.001
Distance measurement (reference measurement)	Data sheet	0.024	Normal	2	0.012
Distance measurement (DUT measurement)	Data sheet	0.024	Normal	2	0.012
Combined uncertainty					0.289
Expanded uncertainty (95% confidence level)					0.578

## DUT characterization

After the successful characterization of the AC with the MAM, the *RL*, G, and radiation pattern measurements of the DUT are done.

### RL measurement

**Figure 12:**
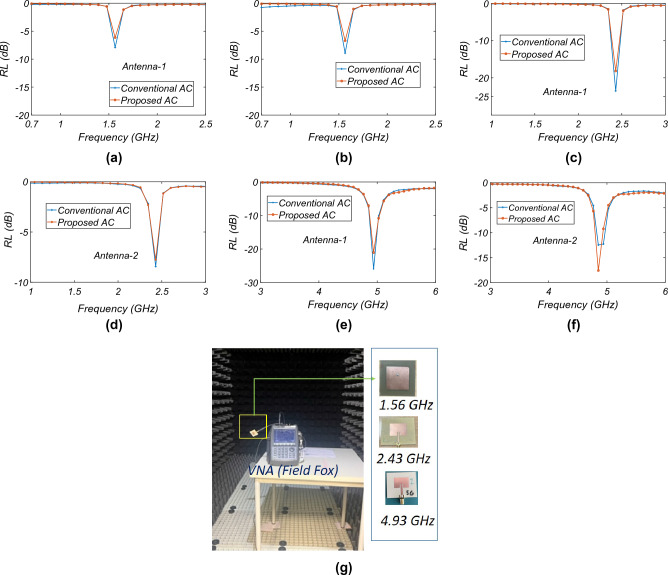
*RL* measurement and comparison: (**a**,**b**) 1.56 GHz, (**c**,**d**) 2.43 GHz, (**e**,**f**) 4.93 GHz, and (**g**) *RL* measurement in conventional AC.

The *RL* of the three microstrip patch antenna samples operating at 1.56 GHz, 2.43 GHz, and 4.93 GHz is measured inside the conventional AC in the author’s premises and the proposed AC. The two units of each sample antenna are fabricated for repeatability and for measurement of the radiation pattern and gain with the two-antenna method measurement procedure, as shown afterward in the literature. Figure 12 shows the *RL* of the fabricated antenna samples. Figure 12g shows the measurement setup in the conventional AC. It is observed from the figures that the *RL* measured with the convention AC matches very well with the proposed AC. The difference in the value of the *RL* in the antenna-1 and antenna-2 at the same operating frequencies is due to fabrication tolerance.

### Realized gain measurement

The DUT’s *G* is measured using the two-antenna method^44,45^. The 2-port standard calibration is done at the ports of connection of the DUT. The DUT is kept in the far field of the Tx antenna. The transmission coefficient ($S_{21}$) between the two antennas in the bore-sight direction is measured at the desired operating frequency. The absolute value of G is given by:

4 $$\begin{aligned} G = \frac{4\times \pi \times r \times \mid S_{21} \mid }{\lambda } \end{aligned}$$

where $\lambda$ is the wavelength of the measurement of the DUT. The measured and simulated G in dBi is tabulated in Table 3. The table shows that the measured G with the proposed AC is within the uncertainty range of 0.578 dB for all the DUT test frequencies, which is a perfect measurement considering the compactness and small size of the AC.

**Table 3:** Measured and simulated G.

DUT test frequency (GHz)	Simulated G (dBi)	Measured G (dBi)	In uncertainty range of 0.578 dB
1.56	4.95	5.4	Yes [4.37, 5.52]
2.43	6.96	7.28	Yes [ 6.38,7.54]
4.93	6.67	6.85	Yes [6.12,7.25]

**Figure 13:**
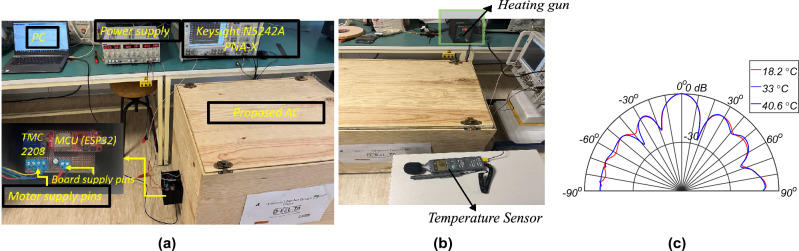
(**a**) The radiation pattern measurement setup, (**b**) thermal analysis setup, (**c**) radiation pattern with varying temperature at 2.5 GHz at $\phi$ = $0^0$.

**Figure 14:**
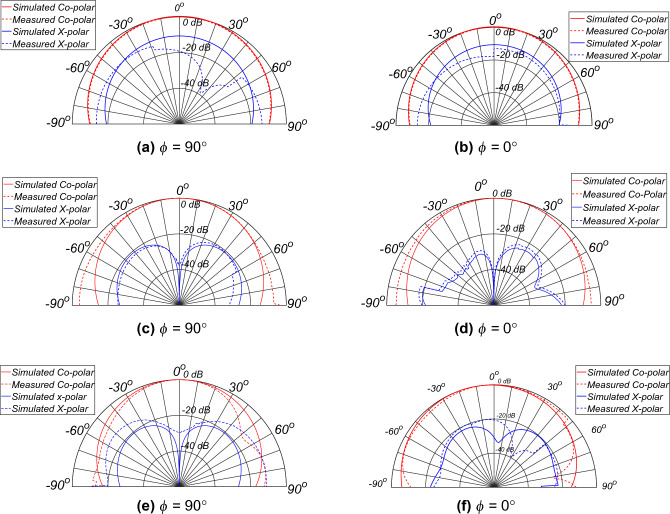
Measured radiation pattern: $\phi$ = 90°: (**a**) 1.56 GHz, (**c**) 2.43 GHz, and (**e**) 4.93 GHz, and $\phi$ = $0^0$: (**b**) 1.565 GHz, (**d**) 2.43 GHz, and (**f**) 4.93 GHz (simulated result: solid line, measured results: dash line).

### Radiation pattern measurement

The automatic radiation pattern measurement system is designed to measure the DUT’s radiation pattern. Figure 13a shows the setup for measuring the radiation pattern of the DUT. Regarding the measurement control process, the programmable network analyzer (PNA) (make: Keysight, model number: N5242A PNA-X) is controlled via MATLAB. The control position system is built using a two-phase stepper motor (NEMA 17) driven using an ultra-silent motor driver IC from ADI Trinamic (TMC2208) and connected to a microcontroller unit (MCU, serial number: ESP32), allowing a rotation from − 90° to 90° with a step of 0.45°.The MCU is connected to the personal computer (PC) via USB, as shown in the figure, which allows it to communicate with MATLAB via Universal Asynchronous Receiver/Transmitter programming. The system requires a 12 V DC power supply for the stepper motor driver, which consumes about 400 mA to operate the NEMA-17 motor. As, the radiation pattern is measured in the closed environment, the thermal test is done. To create the artificial thermal effect, a heat gun (make: Pro’s Kit, model number: SS-969B) is used. The temperature is varied from (temperature before heating) to. The temperature is within the range of safe operation of the stepper motor. The temperature is monitored using a temperature sensor (model number ST-8820). Figure 13b shows the experimental setup and the temperature variation in the AC. The temperature during the test is measured by inserting the probe inside the AC through the small opening that is already created for inserting the cables of the stepper motor. The temperatures is varied from 18.2 °C (normal operation) to 40.6 °C (artificial heating). The radiation pattern of the broadband patch antenna at 2.5 GHz is measured at various temperatures to verify the thermal test as shown in the Fig.13c. To respect the page limit, only the pattern at $\phi$ = 0° is shown. However, the same performance is observed at various frequencies and planes. From the figure, it is observed that there is no significant variation in the radiation pattern with the temperature variation inside the AC. However, care should be taken to not reach a higher temperature to damage the motors and cable parts, and ventilation fans for heat dissipation can be installed in the future. This proves that the proposed AC can handle higher temperatures without any significant or no impact on performance.

Figure 14 shows the co- and cross-polar radiation patterns at 1.56, 2.43, and 4.93 GHz for both the $\phi$ = 90° and $\phi$ = 0° planes. It is observed that the radiation pattern very well matches the simulation, with some deviation at an oblique incidence angle greater than 60°. This is due to the larger *RL* of the MAM above 60° as observed in the MAM, characterising by the simulation and measurement results of the MAM for oblique incidence. This proves that the proposed AC with the 2D MAM can be used for the characterising the antennas in the near future.

## Conclusion

A novel MAM is designed and characterised for 0.7 to 18 GHz, having an overall thickness of 0.012 $\lambda$ where $\lambda$ is calculated at 0.7 GHz. The MAM is fabricated on a transparent ITO+PET substrate. The broadband absorption is achieved by combining three phenomena, i.e., the dipole-like resonance because of the cross-slot on the ITO around a central frequency of 6.5 GHz, the air-gap spacing provided by the styrofoam, and the resistive nature of the sheets. The performance of the MAM for oblique incidence and different polarisation is measured. The proposed MAM is polarisation insensitive due to fourfold symmetry and gives *RL* $\le$ − 10 dB up to $\theta$ = 60°. To prove the conformality of the MAM, the proposed design is a bend with a central angle of 120°. The *RL* remains $\le$ − 10 dB throughout the frequency band, showing the conformal behavior of the proposed MAM. The fabricated MAM is used in designing the AC for the measurement of the radiation pattern of the antenna. The antenna characteristics of the three sample antennas working at 1.56, 2.43, and 4.9 GHz are done. Also, the radiation pattern in varying temperature senario inside the chamber upto 40.6  °C is measured. The measured radiation pattern, gain, and *RL* match the simulation studies. These studies open for the first time the use of MAM in AC design and antenna characterization.

### Supplementary Information

Supplementary Information.

## Data Availability

All data generated or analysed during this study are included in this published article (and its supplementary information files).
